# Occupational Factors Associated with Health-Related Quality of Life in Nursing Professionals: A Multi-Centre Study

**DOI:** 10.3390/ijerph17030982

**Published:** 2020-02-05

**Authors:** María Dolores Ruiz-Fernández, Ángela María Ortega-Galán, Cayetano Fernández-Sola, José Manuel Hernández-Padilla, José Granero-Molina, Juan Diego Ramos-Pichardo

**Affiliations:** 1 Department of Nursing Science, Physiotherapy and Medicine, University of Almería, 04120 Almería, Spain; mrf757@ual.es (M.D.R.-F.); cfernan@ual.es (C.F.-S.); j.hernandez-padilla@ual.es (J.M.H.-P.); jgranero@ual.es (J.G.-M.); 2 Department of Nursing, University of Huelva, 21071 Huelva, Spain; juan.ramos@denf.uhu.es; 3 Associate Researcher, Faculty of Health Science, Universidad Autónoma de Chile, Temuco 4780000, Chile; 4 Adult, Child and Midwifery Department, School of Health and Education, Middlesex University, London NW4 4BT, UK

**Keywords:** nursing, health profession, quality of life, professional quality of life, compassion fatigue, burnout, compassion satisfaction

## Abstract

Background: Nursing professionals are exposed to stressful situations arising from the work context that may affect health-related quality of life (HRQoL). The objective of this study was to analyse the relationship between sociodemographic and work-related variables regarding HRQoL in nursing professionals. Methods: A multi-centre, cross-sectional descriptive design was used. The participants consisted 1521 nurses working in healthcare centres, in both primary care and hospital care, in the eight provinces of the Andalusian Public Health System (APHS), Spain. Sociodemographic and work-related variables were analysed: Compassion fatigue, compassion satisfaction and burnout were measured using the professional quality of life questionnaire (ProQOL), and HRQoL was measured using the SF-12 health questionnaire. Results: Compassion fatigue, burnou, and, to a lesser extent, compassion satisfaction significantly influence the physical and mental components of HRQoL. The simple regression analysis showed that burnout and compassion fatigue were significantly associated with the mental component of HRQoL. Gender influenced the mental component of HRQoL. The rest of the sociodemographic and work-related variables were not significantly related to HRQoL. Conclusion: Work-related stress and repeated contact with situations of suffering influence HRQoL. Health systems must implement programmes to increase the emotional well-being of workers.

## 1. Introduction

Healthcare professionals face situations of intense psychological and emotional involvement in daily practice [1]. Repeated contact with the suffering of patients and organisational aspects of their work context are considered risk factors affecting the psychological and emotional well-being of workers [2,3].

This scenario causes professionals to find themselves in situations of vulnerability [4] that may be conducive to the development of various mental conditions (stress, anxiety, fatigue, exhaustion) related to burnout syndrome and compassion fatigue [5]. Both syndromes have a direct, negative impact on health professionals’ quality of life, well-being and perceived health [6,7]. On the other hand, a positive aspect arising from helping patients is compassion satisfaction, the rewarding feeling that professionals experience when caring for others which encourages them to continue to do so [8]. These three constructs (burnout, compassion fatigue and compassion satisfaction) are encompassed in the concept of Professional Quality of Life (PQoL), described by Stam [9], which refers to the feeling of well-being originating from the balance that individuals perceive between the burden of their profession and the psychological, organisational and interpersonal resources available to them in order to deal with this burden [10]. The ProQoL scale [7] is a useful tool for assessing the positive and negative effects of continued contact with emotionally difficult situations on healthcare professionals. The ProQoL has been used for this purpose in various studies [7,9,11].

In addition, another concept that has become widespread in recent years in relation to the state of health of individuals is Health-Related Quality of Life (HRQoL) [12]. This concept refers to the assessment of individuals’ perceived health, including both objective and subjective aspects [13] of their physical, psychological, social and spiritual well-being [14]. HRQoL is a multidimensional concept, which consists of variables from the physical, mental, social and cultural contexts [15]. Shumaker and Naughton found that the subjective assessment of an individual’s health status affects their physical function and overall state of emotional well-being [16]. 

Exposure to stressors at work and lack of adequate coping resources may affect health professionals’ mental or physical health. This results in reduced HRQoL, which, in turn, results in a reduction in the quality of patient care [17]. According to the existing literature, several components have been assessed and classified as unfavourable by professionals [18], indicating that they may have consequences for the professionals’ perception of their HRQoL [3,4,5,6,7,8,9,10,11,12,13,14,15,16,17,18,19]. These components include characteristics of their work environment (shift, employment status, setting), work-related stress [20], and sociodemographic factors affecting them, such as age, marital status, years of work experience and particularly gender [21]. Women’s health and emotional well-being may be more severely affected than men’s, as work on their jobs may be compounded by household chores and family care [22]. In this sense, it is nursing professionals who are the most fragile and have a high risk of having their HRQoL affected by the characteristics of their work [20,21,22,23]. Their work environment and their experiences with patients negatively affect nurses’ HRQoL [24].

With respect to Spain in particular, over the last decade, adjustments made to the National Health System as a result of the economic crisis have left nursing professionals with a growing sense of worsening working conditions. This translates into understaffing, work overload, decreased time for direct patient care, increased working hours, lack of solidarity with healthcare professionals on the part of managers and decreased salaries [25]. In addition, studies on PQoL have suggested that Spanish nurses have moderate to high levels of compassion fatigue and burnout [26]. In its Ottawa Charter for Health Promotion, the World Health Organization (WHO) recommends that a systematic assessment be made of the impact of changes in the working environment on the perception of health. In addition, research indicates that burnout and compassion fatigue may be determinants of the perception of HRQoL [27]. Despite all this, there are no studies that explore the relationship between these and other work-related variables among Spanish nurses. For this reason, the objective of this study was to analyse the relationship of sociodemographic variables (compassion fatigue, compassion satisfaction and burnout) and work-related variables with HRQoL in nursing professionals in the Spanish public healthcare system.

## 2. Material and Methods

### 2.1. Design and Participants

A multi-centre, cross-sectional descriptive design was used. The sample size necessary was calculated based on the number of nurses who worked at the APHS in 2015 (N = 22,533) with a two-tailed 95% confidence interval, with an accuracy level of 3%, assuming that the expected burnout rate was 32.20% [26]. This resulted in a required minimum sample size of 991 participants. For the selection of the sample, the inclusion criterion established was being an active nursing professional currently working in healthcare services in direct contact with patients. Professionals working in healthcare administration management or in services where there was no direct contact with patients (sterilisation, laboratory, etc.) were excluded. The final sample consisted of 1521 nurses who met the inclusion criterion and agreed to participate.

### 2.2. Instruments

A sociodemographic and work-related data collection sheet was prepared, including the following: Age, gender, marital status (single, married or in civil partnership and separated or widow/er), work environment or setting (primary care, hospital care), employment status (temporary or permanent), work shift (morning shift, rotating without night shifts, rotating with night shifts or fixed night shift), and work experience (years).

PQoL was assessed using the Professional Quality of Life Scale, ProQOL v. IV. [7,8,9]. This questionnaire was translated into Spanish by Morante (2006) and has been used among healthcare professionals [28,29]. This is a self-report questionnaire consisting of 30 items rated on a five-point Likert scale (ranging from 1 = “never” to 5 = “very often”). The scale was divided into three subscales: Compassion fatigue (CF, 10 items), compassion satisfaction (CS, 10 items) and burnout (BO, 10 items). Higher scores on each subscale indicate higher CF, CS and BO values. The following Cronbach’s alpha values were obtained: 0.80 for CF, 0.89 for CS and 0.71 for BO [9].

HRQoL was assessed using the SF-12 Health Questionnaire, made up of 12 items obtained from the SF-36, which is one of the most widely used instruments for HRQoL assessment worldwide [30,31]. The SF-12 provides a health status profile through the assessment of eight dimensions, which are in turn grouped into two components: The physical component (which includes the dimensions of physical functioning, role-physical, bodily pain and general health) and the mental component (which includes the dimensions of mental health, vitality, social functioning and role-emotional). For each of these two components a summary score is obtained that can range from 0 to 100. Higher scores indicate higher levels of HRQoL. Scores over 60 represent a high HRQoL, scores of between 40 and 60 represent a normal HRQoL and scores below 40 represent impaired or low HRQoL. The internal consistency in the Spanish population was found to be 0.7:0.85 for the physical component and 0.78 for the mental component [30,31].

### 2.3. Procedure

Data were collected from January to December 2018 in a total of 12 hospitals, 26 primary care districts, and 10 healthcare management areas (including primary care and hospital care) in Andalusia, Spain. To be able to reach potential participants, the researchers contacted the heads of the healthcare centres to request their collaboration and approval. Once this was obtained, a researcher trained in data collection techniques introduced the study and its objectives to the professionals at each centre, invited them to participate and informed them that their participation was voluntary and anonymous. The subjects who agreed to participate in the study were informed about the objective of the study and were then asked to give their informed consent in writing and to complete the data collection notebook. The estimated completion time was approximately 20 min. Approval was obtained from the Research Ethics Committee of Almería Centro (CEI-27/9/2017). The ethical principles enshrined in the Declaration of Helsinki were observed at all times. The study complied with the national regulations in force concerning data anonymity and confidentiality (Spanish Organic Law 3/2018 on Personal Data Protection and Guarantee of Digital Rights).

### 2.4. Data Analysis

Means, standard deviations, ranges and medians of the quantitative variables were calculated. Absolute values and percentages were calculated for the qualitative variables. A Student’s t-test for independent samples and one-way ANOVA were used for the comparison of means in each of the two dimensions that constitute the HRQoL questionnaire (physical and psychological components) on the bases of each of the sociodemographic and work-related variables analysed, using a 95% significance threshold. For the analysis of correlations between variables, Pearson’s correlation was used. Finally, a simple linear regression analysis was performed between each of the components of the HRQoL (dependent variables) and any significant correlated sociodemographic and work-related variables (independent variables). The corrected coefficient of determination (R2) was assessed in order to study how much of the total variance of the dependent variable was explained by each of the independent variables analysed. Data were processed using the statistical program SPSS Statistics v.25 (IBM Corp, Armonk, NY, USA).

## 3. Results

### 3.1. Descriptive Analyses: Sociodemographic Variables, Work-Related Variable and HRQoL

A total of 1521 nursing professionals participated. Their mean age was 47.3 (SD = 8.4) years of age. Their mean work experience was 22.9 (SD = 9.1) years. The majority of the participants were women (75.5%) and were married or in a civil partnership (72.8%). Of the participants, 54.2% worked in hospitals or specialty care centres, and 43.3% worked in rotating shifts with night shifts. With respect to work-related variables, 59.5% had a stable or permanent employment status, and the majority had more than 20 years of work experience (56.7%). As for PQoL, 64.1% of the participants reported having high levels of CF, 44.5% reported low levels of CS and 58.8% reported having moderate levels of BO. The HRQoL analysis yielded a mean score of 51 (SD = 6.9) for the physical component and a mean score of 48.6 (SD = 8.5) for the psychological or mental component (Table 1).

Table 2 shows the differences in the mean scores for each of the HRQoL components. As can be observed, there were significant gender differences in the mental component, with women’s mean scores being lower than those of men. No gender differences were found for the physical component. No differences were found for any of the HRQoL components according to marital status or work-related variables (workplace, employment status, work shift and professional experience). However, differences were found for both the physical and mental HRQoL components according to the categories of the PQoL variables. As a result, higher levels of CF or BO were associated with lower HRQoL scores, and higher levels of CS resulted in higher HRQoL scores.

### 3.2. Correlations and Linear Regression Analysis

Table 3 shows the correlations between the two HRQoL components and the sociodemographic and work-related variables. A statistically significant negative correlation was observed between the physical component and CF (*r* = −0.13; *p* < 0.001) and BO (*r* = −0.17; *p* < 0.001). A statistically significant positive correlation was observed between the physical component and CS (*r* = 0.05; *p* = 0.045). In other words, for the physical component, lower levels of CF and BO and higher levels of CS were associated with higher levels of HRQoL. On the other hand, a statistically significant correlation was observed between the mental component and gender (*r* = 0.07; *p* = 0.004). In addition, a statistically significant negative correlation was observed between the mental component of HRQoL and the dimensions CF (*r* = −0.28; *p* < 0.001) and BO (*r* = −0.33; *p* < 0.001). The correlation between the mental component and CS was found to be positive (*r* = 0.08; *p* = 0.002). This means that lower scores for CF and BO and higher CS scores indicate higher scores for the mental component of HRQoL.

The simple linear regression analysis for the physical component of HRQoL revealed that these three dimensions of PQoL significantly influenced this component: CF (*B* = −0.110; *p* < 0.001), CS (*B* = 0.048; *p* = 0.045) and BO (*B* = −0.223; *p* < 0.001). The dimensions with the greatest weighting were BO (*R*^2^ = 0.029) and CF, which explained 2.9% and 1.5% of the variance of the dependent variable (physical component), respectively (Table 4).

Table 5 shows the linear regression analysis for the mental component of HRQoL. Gender significantly influenced the mental component of HRQoL (*B* = 1.442; *p* = 0.004), as did CF (*B* = −0.303; *t* < 0.001), CS (*B* = 0.092; *p* = 0.002) and BO (*B* = −0.521; *t* < 0.001). However, the factors with the greatest weighting were BO (*R*^2^ = 0.105) and CF (*R*^2^ = 0.079), which explained 10.5% and 7.9% of the variance of the variable (mental component), respectively (Table 5).

## 4. Discussion

The objective of this study was to analyse the influence of certain sociodemographic and work-related variables on the HRQoL of nursing professionals. CF, BO and, to a lesser extent, CS, were variables that influenced HRQoL, particularly the mental component of this construct. In addition, gender was related to the mental component of HRQoL. The rest of the soci-demographic and work-related variables were not significantly related to HRQoL.

In this study, the scores for HRQoL, which was measured with the SF-12 questionnaire, were, on average, higher in the physical dimension than in the mental dimension, which is consistent with previous studies [32,33]. However, in a study by Sarafis (2016) with nurses working in nursing homes, the mean scores for both dimensions were similar to each other and lower than those found in the present research [34]. Our study was conducted with a very large sample, covering the entire range of services in which patients and their families are directly cared for at the APHS. The differences in HRQoL observed between this study and previous studies might be influenced by the fact that working in very specific services and in situations of extreme vulnerability affects professionals’ mental health [35,36].

The work context and the experiences with patients to which health workers are subjected may affect how they perceive their general well-being and health [14,15,16,17,18,19,20,21,22,23,24,25,26,27,28,29,30,31,32,33,34,35,36,37]. The results obtained in this research show how the three dimensions of PQoL (CF, BO and CS) influence HRQoL. This is in line with the results of studies conducted in other countries, such as Israel [38]. Studies showing the influence of burnout and occupational stressors on nurses’ HRQoL have also been conducted elsewhere, e.g., China [17]. It therefore appears that emotional stressors related to nursing practice may be a major influence on these professionals’ HRQoL, regardless of the sociocultural context in which the profession is practised. However, specific studies with samples originating from several countries are needed to explore this issue.

The close emotional bond that may eventually develop with individuals and their families throughout their lives is a determining factor that affects PQoL [39,40]. Exposure to certain situations of suffering and distress in the work environment often results in professionals becoming vulnerable to particular syndromes, such as CF [41]. In this study, CF levels were high in a large percentage of the sample and were significantly related to the two components of HRQoL, particularly the mental component. Caring for sick individuals and, above all, empathising with them and trying to alleviate their suffering may be detrimental to and cause a number of negative effects on health [42]. Duarte (2016) studied how affective empathy might be a risk for presenting with CF, while the positive components of self-compassion, such as kindness and humility, are protective factors against CF [43].

However, according to our results, CS was only weakly associated with HRQoL. Feeling satisfied, in terms of compassion with the care and assistance delivered to others, does not necessarily imply feeling better or that one’s quality of life is not affected, as pointed out in other studies [8].

Other studies have shown that BO and exposure to stressors in the work environment and in the occupational structure have a negative impact on HRQoL, in line with the results of this research [6,7,8,9,10,11,12,13,14,15,16,17,18,19,20,21,22,23,24,25,26,27,28,29,30,31,32,33,34,35,36,37,38,39,40,41,42,43,44]. Personal tensions, feelings of self-efficacy and personal exhaustion generated by the work environment have a greater effect on the mental component of HRQoL. In turn, factors more closely related to the work context and the workload affect the physical dimension more [17].

The data from this study indicate that the gender variable is related to the mental component of HRQoL, but not to its physical component. Other studies related gender to HRQoL, principally with the mental or psychological component [22,23,24,25,26,27,28,29,30,31,32,33,34,35,36,37,38,39,40,41,42,43,44,45]. Additionally, some studies found that female participants experience a greater presence of psychosomatic symptoms than their male counterparts [46]. Moreover, several studies showed that women are more likely to express concern for others and to exhibit empathic responses [47]. Among nursing professionals, this difference continues to be influenced primarily by social role assumptions rather than by differences in empathic skills [48]. It should be added that women usually perceive higher levels of workload due to the difficulties that they face in attaining a work-life balance [23]. However, other studies that have explored gender differences in the work context found that men experience greater exhaustion and are more likely to become depersonalised [49]. This morbidity may be due to the type of coping strategy used, with women using a coping mechanism which focuses on the emotional component in a bid to regulate the emotions that arise rather than addressing the problem directly [50].

In our study, other sociodemographic and work-related variables were not shown to be significantly related to HRQoL. These results stand in contrast to other studies, which have determined that characteristics of the work environment, such as work shift, employment status [21] and experience in years, have an impact on HRQoL, as do the sociodemographic characteristics of professionals, such as age or marital status [22].

One of the limitations of this study is that it involved a sample of aged nurses with extensive work experience, with more than half of them having more than 20 years of work experience. This data may have skewed the results obtained. Nevertheless, it should be kept in mind that this was a representative sample of the population of the nurses currently working at the APHS. In addition, this was a very varied sample of professionals working at different care levels and in different regions and healthcare settings. Second, this was a descriptive study, and the variables analysed were prognostic indicators. However, no cause-and-effect relationships were established. To this end, it would be necessary to conduct a longitudinal study and establish the strength of the associations of the variables analysed. Additionally, social desirability bias in the completion of questionnaires cannot be ruled out in this study.

## 5. Conclusions

Nursing professionals had lower scores on the mental component of HRQoL than on the physical component. Work-related and sociodemographic variables were not associated with HRQoL, except for gender, which was significantly associated with the mental component of HRQoL. CF, BO and, to a lesser extent, CS influenced the physical and mental components of HRQoL, particularly the mental component. Further research should be conducted on the role that these variables may play in the HRQoL of healthcare professionals, as these variables are more influential than others that are specifically work-related (workplace, employment status, work shift and work experience).

Healthcare service managers in Spain have made efforts to improve the work-life balance of nurses by implementing strategies, such as improved rotating shifts, in an attempt to increase their quality of life. However, they tend to attach little importance to the effect that continued contact with the suffering of the individuals they care for has on nurses, despite the fact that studies such as ours indicate that this is an influential factor in nurses’ well-being. Health system managers should therefore implement health promotion and prevention interventions among nurses to improve their HRQoL and reduce their risk of absenteeism or resignation. In this sense, we believe that interventions centred on the cultivation of or training in compassion might be a core element in such interventions [51].

Similarly, it could be useful to introduce the content into nursing training programmes on coping, emotion management and/or compassion and self-compassion cultivation required to enable nurses to address the ongoing suffering of others effectively from the beginning of their careers. These interventions may also be usefully implemented in countries other than Spain, taking into consideration that occupational stressors (burnout and compassion fatigue) seem to influence HRQoL regardless of the country or cultural setting. In any case, further studies are needed to assess the effectiveness of the interventions proposed in order to alleviate burnout and compassion fatigue, and to improve nurses’ HRQoL.

## Figures and Tables

**Table 1:** Descriptive sociodemographic, work-related and Health-Related Quality of Life (HRQoL_ data of the nurses (*n* = 1521).

Characteristics	% (*n*)	Mean ± SD	Range	Median
**Age**		47.3 ± 8.4	23–64	47
**Gender**				
Male	24.5 (1148)			
Female	75.5 (373)			
**Marital status**				
Single	15.9 (241)			
Married/civil partnership	72.8 (1107)			
Separated or widow/er	11.3 (173)			
**Workplace**				
Primary care centre	45.8 (697)			
Hospital or specialty care centre	54.2 (824)			
**Employment status**				
Temporary/replacement/unstable	40.5 (616)			
Stable/permanent	59.5 (905)			
**Work shift**				
Fixed morning shift	32.1 (488)			
Rotating without night shifts (mornings/evenings)	23.9 (364)			
Rotating with night shifts	43.3 (659)			
Fixed night shift	0.7 (10)			
**Experience as a nurse (in years)**		22.9 ± 9.1	0.42–46	23
<10 years	9.6 (146)			
10–20 years	33.7 (513)			
>20 years	56.7 (862)			
**Compassion fatigue (ProQoL)**		20.7 ± 7.9	1–42	21
Low	6.6 (100)			
Moderate	29.3 (446)			
High	64.1 (975)			
**Compassion satisfaction (ProQoL)**		35.5 ± 7.4	16–50	35
Low	44.5 (677)			
Moderate	32.7 (497)			
High	22.8 (347)			
**Burnout (ProQoL)**		23.4 ± 5.3	4–41	24
Low	17.6 (268)			
Moderate	58.8 (894)			
High	23.6 (359)			
**Physical component (SF-12)**		51.0 ± 6.9	19.7–66.6	52.3
**Mental component (SF-12)**		48.6 ± 8.5	10.5–63.1	50.9

SD = standard deviation; ProQoL = Professional Quality of Life.

**Table 2:** Differences in means in HRQoL scores according to sociodemographic and work-related variables of the nurses (*n* = 1521).

Characteristics		Physical Component (SF-12)		Mental Component (SF-12)	
Variables	% (*n*)	Mean ± SD		Mean ± SD	
**Gender**					
Male	24.5 (1148)	51.3 ± 6.6	*t* = −1.15 *p* = 0.25	49.6 ± 7.8	*t* = −2.86 *p* = 0.004
Female	75.5 (373)	50.9 ± 7.0		48.2 ± 8.7	
**Marital status**					
Single	15.9 (241)	51.2 ± 7.1	*F* = 1.33 *p* = 0.27	48.2 ± 8.6	*F* = 0.88 *p* = 0.41
Married/Civil partnership	72.8 (1107)	50.8 ± 6.9		48.7 ± 8.4	
Separated or widow/er	11.3 (173)	51.7 ± 6.2		47.9 ± 8.5	
**Workplace**					
Primary care centre	45.8 (697)	50.9 ± 6.8	*t* = −0.50 *p* = 0.62	48.4 ± 8.6	*t* = −0.46 *p* = 0.65
Hospital or specialty care centre	54.2 (824)	51.1 ± 7.0		48.6 ± 8.4	
Employment status					
Temporary/replacement/unstable	40.5 (616)	50.9 ± 6.9	*t* = −0.14 *p* = 0.89	48.7 ± 8.1	*t* = 0.59 *p* = 0.70
Stable/permanent	59.5 (905)	51.0 ± 6.9		48.5 ± 8.8	
**Work shift**					
Fixed morning shift	32.1 (488)	50.8 ± 7.1	*F* = 0.66 *p* = 0.58	48.5 ± 8.8	*F* = 0.72 *p* = 0.54
Rotating without night shifts (mornings/evenings)	23.9 (364)	51.2 ± 6.7		48.1 ± 8.3	
Rotating with night shifts	43.3 (659)	51.0 ± 6.8		48.8 ± 8.4	
Fixed night shift	0.7 (10)	53.3 ± 5.7		50.7 ± 5.7	
**Experience as a nurse (in years)**					
<10 years	9.6 (146)	51.6 ± 6.3	*F* = 0.76 *p* = 0.47	48.8 ± 7.9	*F* = 0.43 *p* = 0.65
10–20 years	33.7 (513)	51.1 ± 7.1		48.3 ± 8.6	
>20 years	56.7 (862)	50.8 ± 6.9		48.7 ± 8.5	
**Compassion fatigue (ProQoL)**				
Low	6.6 (100)	53.7 ± 4.6	*F* = 15.08 *p* < 0.001	53.3 ± 6.7	*F* = 38.99 *p* < 0.001
Moderate	29.3 (446)	51.8 ± 6.9		50.4 ± 7.3	
High	64.1 (975)	50.4 ± 7.0		47.2 ± 8.8	
**Compassion satisfaction (ProQoL)**					
Low	44.5 (677)	50.9 ± 6.4	*F* = 5.09 *p* = 0.006	48.5 ± 8.2	*F* = 11.35 *p* < 0.001
Moderate	32.7 (497)	50.4 ± 7.4		47.5 ± 8.7	
High	22.8 (347)	51.9 ± 6.9		50.3 ± 8.5	
**Burnout (ProQoL)**					
Low	17.6 (268)	52.9 ± 5.9	*F* = 16.77 *p* < 0.001	52.1 ± 6.9	*F* = 69.47 *p* < 0.001
Moderate	58.8 (894)	50.9 ± 6.9		49.1 ± 7.8	
High	23.6 (359)	49.8 ± 7.3		44.6 ± 9.7	

SD = standard deviation; *t* = Student’s *t*; *F* = ANOVA; *p* = level of statistical significance.

**Table 3:** Bivariate correlations between HRQoL and sociodemographic and work-related variables of the nurses (*n* = 1521).

Variables	Physical Component (SF-12)	Mental Component (SF-12)
Age	*r* = −0.04	*r* = −0.02
	*p* = 0.14	*p* = 0.41
Gender	*r* = 0.03	*r* = 0.07
	*p* = 0.27	*p* = 0.004
Marital status (Married/Not married)	*r* = 0.04	*r* = −0.03
	*p* = 0.13	*p* = 0.20
Workplace (Primary care/Specialty care)	*r* = 0.01	*r* = 0.01
	*p* = 0.62	*p* = 0.65
Employment status (stable/unstable)	*r* = 0.03	*r* = −0.01
	*p* = 0.89	*p* = 0.70
Work shift (with night shifts/without night shifts)	*r* = 0.004	*r* = 0.03
	*p* = 0.86	*p* = 0.30
Professional experience (years)	*r* = −0.04	*r* = 0.002
	*p* = 0.11	*p* = 0.94
Compassion fatigue	*r* = −013	*r* = −0.28
	*p* < 0.001	*p* < 0.001
Compassion satisfaction	*r* = 0.05	*r* = 0.08
	*p* = 0.045	*p* = 0.002
Burnout	*r* = −0.17	*r* = −0.33
	*p* < 0.001	*p* < 0.001

*r* = Pearson’s correlation; *p* = level of statistical significance.

**Table 4:** Simple linear regression coefficients for the physical component of HRQoL. Nursing professionals (*n* = 1521).

Variables	Constant (SE)	*B* (SE)	Beta	*t*	*p*	Corrected *R*^2^
Compassion fatigue	53.284 (0.494)	−0.110 (0.022)	−0.126	−4.959	<0.001	0.015
Compassion satisfaction	49.292 (0.866)	0.048 (0.024)	−0.051	2.008	0.045	0.002
Burnout	56.215 (0.791)	−0.223 (0.033)	−0.171	−6.763	< 0.001	0.029

SE = standard error; *t* = Student’s *t*; *p* = level of statistical significance; *R*^2^ = coefficient of determination.

**Table 5:** Simple linear regression coefficients for the mental component of HRQoL. Nursing professionals (*n* = 1521).

Variables	Constant (SE)	*B* (SE)	Beta	*t*	*p*	Corrected *R*^2^
Gender	46.759 (0.665)	1.442 (0.505)	−0.073	2.857	0.004	0.005
Compassion fatigue	54.839 (0.588)	−0.303 (0.027)	−0.281	−11.426	<0.001	0.079
Compassion satisfaction	45.282 (1.064)	0.092 (0.029)	0.080	3.142	0.002	0.006
Burnout	60.761 (0.935)	−0.521 (0.039)	−0.325	−13.378	<0.001	0.105

SE = standard error; *t* = Student’s *t*; *p* = level of statistical significance; *R*^2^ = coefficient of determination.
